# Clinical Symptoms and Risk Factors in Cerebral Microangiopathy Patients

**DOI:** 10.1371/journal.pone.0053455

**Published:** 2013-02-05

**Authors:** Sandra Okroglic, Catherine N. Widmann, Horst Urbach, Philip Scheltens, Michael T. Heneka

**Affiliations:** 1 Department of Neurology, Clinical Neuroscience Unit, University of Bonn, Bonn, Germany; 2 Department of Radiology, Neuroradiology Unit, University of Bonn, Bonn, Germany; 3 Alzheimer Center and Department of Neurology, VU University Medical Center, Amsterdam, The Netherlands; University of Münster, Germany

## Abstract

**Objective:**

Although the clinical manifestation and risk factors of cerebral microangiopathy (CM) remain unclear, the number of diagnoses is increasing. Hence, patterns of association among lesion topography and severity, clinical symptoms and demographic and disease risk factors were investigated retrospectively in a cohort of CM patients.

**Methods:**

Patients treated at the Department of Neurology, University of Bonn for CM (*n = *223; 98*m*, 125*f*; aged 77.32±9.09) from 2005 to 2010 were retrospectively enrolled. Clinical symptoms, blood chemistry, potential risk factors, demographic data and ratings of vascular pathology in the brain based on the Wahlund scale were analyzed using Pearson's chi square test and one-way ANOVA.

**Results:**

Progressive cognitive decline (38.1%), gait apraxia (27.8%), stroke-related symptoms and seizures (24.2%), TIA-symptoms (22%) and vertigo (17%) were frequent symptoms within the study population. Frontal lobe WMLs/lacunar infarcts led to more frequent presentation of progressive cognitive decline, seizures, gait apraxia, stroke-related symptoms, TIA, vertigo and incontinence. Parietooccipital WMLs/lacunar infarcts were related to higher frequencies of TIA, seizures and incontinence. Basal ganglia WMLs/lacunar infarcts were seen in patients with more complaints of gait apraxia, vertigo and incontinence. Age (*p = *.012), arterial hypertension (*p<*.000), obesity (*p<*.000) and cerebral macroangiopathy (*p = *.018) were positively related to cerebral lesion load. For increased glucose level, homocysteine, CRP and D-Dimers there was no association.

**Conclusion:**

This underlines the association of CM with neurological symptoms upon admission in a topographical manner. Seizures and vertigo are symptoms of CM which may have been missed in previous studies. In addition to confirming known risk factors such as aging and arterial hypertension, obesity appears to increase the risk as well. Since the incidence of CM is increasing, future studies should focus on the importance of prevention of vascular risk factors on its pathogenesis.

## Introduction

C*erebral microangiopathy* (CM), also *cerebral small vessel disease*, is diagnosed with increasing frequency. Improved neuroimaging techniques, aging, as well as dietary and life-style changes leading to a higher incidence of vascular risk factors may play an important role. The exact reasons however remain unclear. CM refers to pathological changes in small brain vessels, including small arteries, arterioles, capillaries and small veins [1]. It is associated with *white matter lesions* (WMLs), lacunar infarcts, and, more recently, microbleeds [2]–[4]. CM manifests itself in manifold clinical symptoms including gait disorders, [5] urinary disturbances, [6] depression [7] and cognitive decline [8]–[10]. Because CM patients are such a heterogeneous group, clinicians may not consider this diagnosis and may miss important opportunities for intervention. Hence, study of the clinical manifestations of CM is warranted.

In addition, there is a lack of clarity about potential risk factors: arterial hypertension and age are recognized as major risk factors for CM, [11], [12] yet it is not certain whether diabetes, obesity and hyperlipidemia pose additional risks [13]–[17]. Studies of associations between blood chemistry markers (d-dimers, homocysteine and CRP) and CM have also yielded inconsistent results [18]–[23].

For these reasons we retrospectively examined clinical symptoms, blood chemistry, potential risk factors and demographic data in a large cohort of patients looking for patterns related to vascular pathology in the brain. First, lacunar infarcts and white matter lesions were rated according to topography and severity in CM patients. Then clinical symptoms were divided into logical classes a priori for purposes of analysis: stroke-related-symptoms, transient ischemic attack-symptoms, seizures, gait apraxia, vertigo, progressive cognitive decline and incontinence. The main question of interest was whether lesion topography and severity were, indeed, associated with the manifestation of particular clinical symptoms, and whether they were associated with particular demographic or disease risk factors (e.g., age, gender, heart disease and obesity).

## Materials and Methods

### Ethics Committee Approval

No experimental or new protocols were done. All patients were diagnosed and treated at the hospital according to national guidelines and agreements and not for the purpose of any study. Data were handled anonymously and analyzed only in group form so that no information can be used to identify any individuals in the sample.

Following local requirements, a description of the study was sent to the chair of the local ethics committee for review. It was confirmed by letter that no approval was required from the local ethics committee, signed by Prof. Dr. K. Racké, Chair of the Rheinische Friedrich-Wilhelms-Universität Medical Faculty Ethics Commission, Biomedizinisches Zentrum, Sigmund-Freud-Str. 25, 53105 Bonn, Germany (Letter Reference Number 210/12). The basis for this decision referred to in this letter was §15 of the medical professional code of the North Rhine Medical Association.

*Inclusion* requirements were (1) 45–95 years of age; (2) CM detected by typical radiological (CT/MRT) findings such as WMLs of any degree or lacunar infarcts using Wahlund et al.'s categorization [24]. *Exclusion* criteria comprised (1) presence of severe illnesses (major cerebral bleeding due to secondary causes such as vascular malformation or trauma, coma, cardiac or hepatic failure, cancer or other relevant systemic diseases); (2) severe unrelated neurological diseases; (3) leukoencephalopathy of non-degenerative origin (immunologic–demyelinating, metabolic, toxic, infectious, genetic, other); (4) severe psychiatric disorders, (5) those over 95 years of age were excluded due to the high numbers of comorbidities which may confound results.

### Subjects

Medical records were perused for diagnosed cases of CM among all patients treated at the stroke unit (5%), neurological ward (80%) or outpatient clinic (15%) of the Department of Neurology Bonn, University Medical Center. Cases were identified by evidence of CM in neuroradiological imaging. In total 2509 records were rated. Of the 276 patients diagnosed with CM, 53 did not fulfill inclusion criteria. A total of 223 patients were retrospectively enrolled in this study.

### Clinical presentation

Symptoms at clinical presentation were divided a priori into eight logical categories: seizures, vertigo, gait apraxia, incontinence, stroke-related symptoms, transient ischemic attack (TIA)-symptoms, progressive memory complaint/dementia, and other nonspecific reasons for undergoing cranial neuroimaging study (i.e., WML or lacunar infarcts were found incidentally).

Seizures were defined according to the International League against Epilepsy (ILAE) as transient occurrence of signs and/or symptoms due to abnormal excessive or synchronous neuronal activity in the brain and were confirmed by a physician or a family member (thus, postictal paresis could be well distinguished from TIA). Gait apraxia was considered as inability to walk, unrelated to features as incoordination, sensory loss and failure to comprehend simple commands. Stroke-related symptoms were defined as continuous symptoms associated with stroke according to National Institute of Neurological Disorders and Stroke (NINDS) criteria which were neuroradiologically corroborated. TIA-symptoms were defined as symptoms associated with stroke according to NINDS criteria regressing within 24 hours with no radiological correlation. Progressive memory complaint was defined as progressive memory loss not affecting daily function. Dementia was defined according to ICD-10. Vertigo was specified as a subtype of dizziness, defined as an illusion of movement. Vertigo of non-central origin such as peripheral vestibular or cardial causes were ruled out by careful history taking and with the assistance of otolaryngologic as well as internal medicine consultants [35].

Incontinence was defined as any involuntary leakage of urine. Incontinence was recorded by taking the medical history or by diagnosis during the stay in the clinic. Non-central causes of incontinence were eliminated by careful differentiation in the medical history.

### Clinical Workup

The clinical measures examined were blood chemistry, cardiovascular assessment, clinical tests and physiological recordings. Chemical screenings included coagulation factors, serum values for glucose, HbA1c, CRP, cholesterol, triglycerides, LDL, HDL, and kidney function. Reference values for blood chemistry screenings were taken from the Institute of Clinical Chemistry and Pharmacology at the University of Bonn [5]. Cardiovascular assessment consisted of systolic and diastolic blood pressure, heart rate and mean arterial pressure (MAP). Norm values used were taken from WHO-criteria [5]. Clinical tests as well as physiological recordings comprised carotid duplex, EKG, EEG and Mini Mental Status Examination.

### Risk factors

Among the cardiovascular risk factors included in this examination were *heart disease*, *arterial hypertension, diabetes, hypercholesterolemia*, *obesity*, and *cerebral macroangiopathy*. Heart disease was defined as cardiac arrhythmia, coronary heart disease, heart attack and heart failure. Arterial hypertension was defined as elevated blood pressure over 140/90 mmHg [5]. Diabetes was diagnosed by any of the following criteria: Fasting plasma glucose level ≥7.0 mmol/L (126 mg/dL), plasma glucose ≥11.1 mmol/L (200 mg/dL) two hours after a 75 g oral glucose load as in a glucose tolerance test, symptoms of hyperglycemia and casual plasma glucose ≥ 11.1 mmol/L (200 mg/dL), glycated hemoglobin (Hb A1C) ≥6.5% [5]. *Hypercholesterolemia* was defined as cholesterol level greater than 5 mmol/l [5]. *Obesity* was defined by a body-mass-index (BMI) >25 [5]. *Cerebral Macroangiopathy* was defined as the result of thickening and hardening of the walls of the large arteries in the brain [5].

### Electroencephalography

Ten to twenty minutes of EEG were recorded using the international 10–20 system with additional T1 and T2 electrodes. Linked ear-lobe electrodes were used as reference (A1 and A2). Data was sampled at a rate of 256 Hz using an anti-aliasing lowpass filter. Segments containing artefacts such as eye movements, eye-blinks, other movement artefacts or drowsiness were identified and excluded from further analyses by consensual evaluation of a board-certified EEG expert, blinded for the group membership of the cases. The first five minutes of awake, resting, eyes closed and artefact free EEG segments were selected for further analysis. Power spectra were calculated for consecutive 4-second-windows (1024 samples each) were calculated for each electrode contact, and absolute spectral band power for conventional EEG frequency bands (delta: 0.5–4 Hz; theta: 4–8 Hz; alpha: 8–13 Hz; beta: 13–20 Hz; gamma: 20–40 Hz) were averaged across different windows.

### CT/MRI Scanning

All patients were thoroughly examined and underwent neuroimaging (CT/MRT) within 24 hours after admission. All original scans of magnetic resonance imaging (MRI) or (if not available) computer tomography (CT) were examined for WMLs and lacunar infarcts. The MRI equipment used operated at 3T. The protocol included the following sequences: T1-weighted, T2-weighted and FLAIR, coronal and sagittal plane and slice thickness were 5 mm. Different CT scanners were used. Slice thicknesses of CT scans varied from 2.5–10 mm.

### Imaging analysis

Brain scans were blindly graded by a trained rater and reevaluated by two readers for the study, according to Wahlund, et al.'s rating scale for white matter changes and lacunar infarcts, which is validated for both MRI and CT scans [24]. Scans were searched for white matter lesions and lacunar infarcts. White matter changes on MRI were defined as ill-defined hyperintensities greater or equal 5 mm on both T2 and PD/FLAIR images, and on CT as ill-defined and moderately hypodense areas of greater or equal 5 mm. Lacunes were defined as well-defined areas of >2 mm with attenuation (on CT) or signal characteristics (on MRI) the same as cerebrospinal fluid. If lesions with these characteristics were < = 2 mm, they were considered perivascular spaces, except around the anterior commissure, where perivascular spaces can be large. Grading was carried out for 5 distinct brain areas (1) frontal-, 2) parietooccipital, 3) temporal-, 4) infratentorial brainstem and 5) the basal ganglia), according to the protocol. According to Wahlund and colleagues, the first four brain regions, a grade of zero means no lesions, grade 1 means focal lesions, grade 2 means confluent lesions, grade 3 means diffuse lesions. The grading of the basal ganglia was conducted slightly different: 0 =  No lesions, 1 =  one focal lesion, 2 =  more than one focal lesion, 3 =  confluent lesions [24]. A grade of zero could not be given, by definition, since all patients showed some extent of WML. For statistical analysis, patients were grouped according to the maximum white matter grading regardless of topography (Group 1: maximum grade of 1, Group 2: maximum grade of 2, and Group 3: maximum grade of 3).

### Statistical analysis

For all statistical tests the significance level was set at 5%. Statistical analysis was performed using SPSS for Windows (17.0) [25]. Patients were divided into five age groups in order to evaluate age-related effects: 45–55, 56–65, 66–75, 76–85, and 86–95 years of age. Nonparametric data was analyzed via Pearson's chi square test; parametric data was analyzed by one-way ANOVA. Correction for the greater number of female patients was done using a trend test.

## Results

A total of 223 patients (98*m*, 125*f*) with a mean age of 77.32±9.09 *SD* years were retrospectively enrolled in this study.

### Symptoms associated with CM at admittance

**Figure 1:**
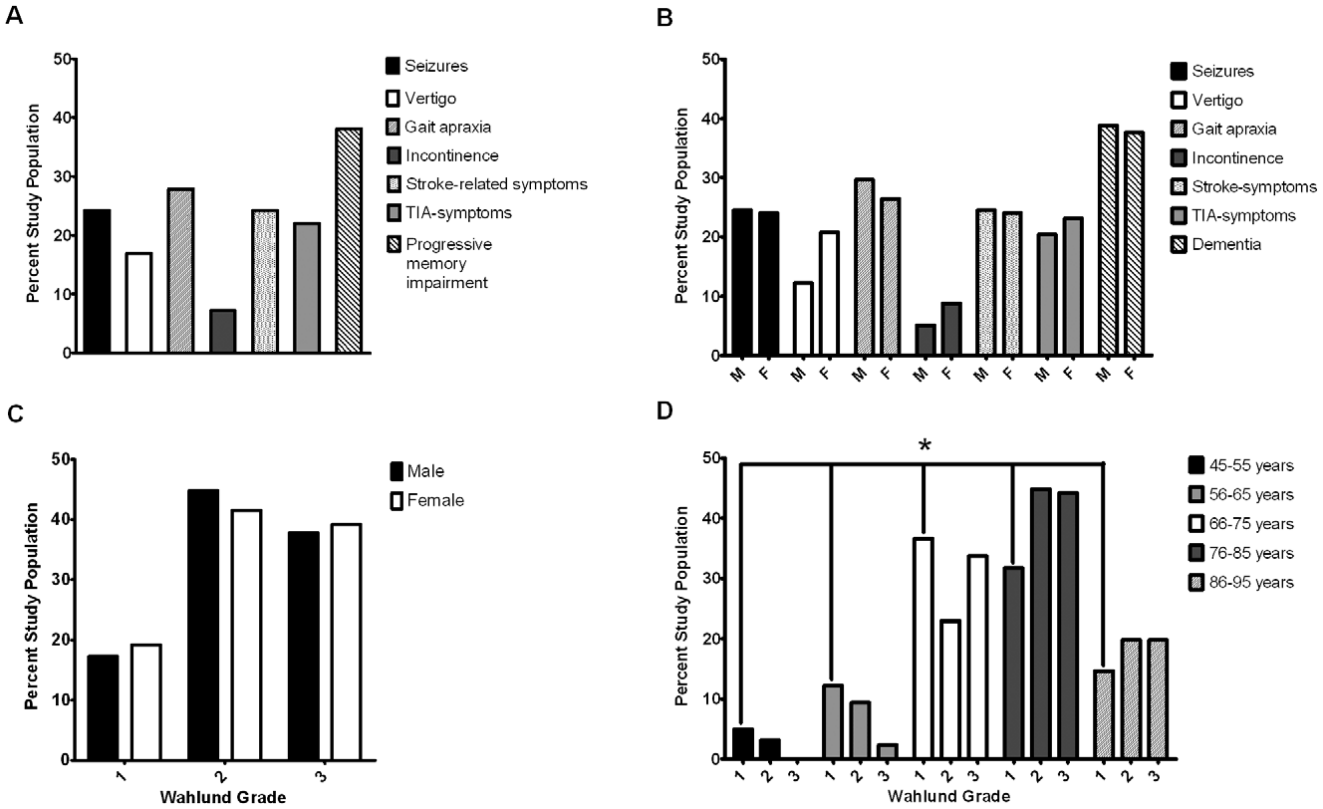
Neurological symptoms at admittance. The symptoms at admittance are described as (**A**) total prevalence in study population (*n* = 223) and (**B**) their relative frequency according to gender (*M*  =  male, *F*  =  female). The symptoms could co-occur in patients and are therefore not mutually exclusive. After correcting for the greater number of female subjects included, all symptoms were equally distributed across genders. (**C**) Equivalent distribution of white matter lesion severity across genders. (**D**) Age influences the severity-grading of WML/lacunar infarcts. Mean age of patients was 77.32±9.09 years. The highest prevalence of WML/lacunar infarcts was found in patients 75–85 years of age, representing 42% of the entire study population. There was an age effect on lesion severity (described by Wahlund, et al., 2001) of CM (One-way, ANOVA, *p = .012).

Of the symptoms associated with CM, progressive memory impairment was the most frequent (38.1%), followed by gait apraxia (27.8%), stroke-related-symptoms and seizures (24.2%), TIA-symptoms (22%), vertigo (17%) and incontinence (7.2%) (Fig. 1A). A mere 7% of all patients were admitted for other reasons. After correcting for the greater number of females by trend test, all symptoms were about equally distributed across genders with the exception of vertigo and incontinence that tended to be more frequent in female patients (Fig. 1B).

### Distribution of WML Grades/lacunar infracts and dependence on gender and age

Around 20% of patients revealed grade 1 lesions, 40% manifested grade 2 lesions and 39% presented with grade 3 lesions. While gender showed no significance (Fig. 1C), age had a strong influence on the severity of WML/lacunar infarcts (Oneway Anova p = 0.12). From the five age cohorts analyzed, patients aged 76–85 years represented 42% of the entire study population compared to 29.6% in the second largest group comprising 66–75 year olds (Fig. 1D). Of note, patients aged 45–55 and 56–65 years displayed mainly grade 1 and 2 WMLs/lacunar infarcts with only a minor number of grade 3 cases. This pattern reversed completely in the older age cohorts, with greater grade 2 and grade 3 being most frequent in patients aged from 66–75 years, and grade 3 dominating in those 76 and older.

### Distribution and Topography of Symptoms

Brain regions including the frontal, temporal and parietooccipital lobe, the basal ganglia and infratentorial brain areas, were analyzed for their WML/lacunar infarct load (Table 1). The vast majority (96.5%) of patients suffering from progressive memory dysfunction exhibited lesions in the frontal lobe. Similarly, 96% of patients with stroke-symptoms showed lesions in this lobe. Patients displaying TIA-symptoms frequently also had with lesions in the parietooccipital (94%) and frontal (92%) lobes, whereas all patients with urinary incontinence showed lesions in the parietooccipital lobe. Vertigo was associated with lesions in the frontal lobe (90%) and, to a lesser extent, with lesions in the basal ganglia and parietooccipital lobe. Patients admitted with seizures revealed a high lesion load in the frontal and parietooccipital lobes (92%), but were almost free from infratentorial WMLs/lacunar infarcts. EEG recordings of these patients showed a direct relation to lesion severity, as dysrhythmic EEGs were more often correlated with a high lesion load (*p = *.003; Pearson's chi square).

**Table 1:** Lesion topography in relation to the main neurological symptoms and severity.

	Lesions according to symptoms (%)				
Clinical Symptoms	Frontal lobe	Parieto-occipital lobe	Temporal lobe	Infratentorial	Basal ganglia
Total in study population	93.3	89.7	51.6	22.4	64.1
Dementia	96.5	89.4	58.8	22.4	68.2
Stroke-Symptoms	96.3	83.7	51.9	22.2	57.4
TIA-Symptoms	91.8	93.9	53.1	28.6	63.3
Incontinence	93.9	100	57.6	24.2	72.7
Vertigo	94.7	76.3	42.1	26.3	76.3
Seizure	90.7	90.7	55.6	14.8	63.0
Gait apraxia	95.2	87.1	51.6	21.0	71.0

The occurrence of main clinical symptoms at admittance was analyzed in relation to WML/lacunar infarct localization in various brain regions, including the frontal, parietooccipital and temporal lobe, infratentorial areas and basal ganglia. Data are given as the frequency of Wahlund Grades (1, 2, and 3) found in each brain region.

Furthermore, gait apraxia appeared predominantly in those with frontal lobe or basal ganglia lesions. A comparison of lesion severity among brain regions rendered an almost equal distribution of focal, confluent and diffuse lesions in the frontal and parietooccipital lobe (Table 2). While the temporal and infratentorial areas showed mostly focal lesions, basal ganglia lesions were mostly of confluent nature.

**Table 2:** Relation of severity of lesions and topography.

	Grades according to brain topology (%)				
Wahlund Grade	Frontal lobe	Parieto-occipital lobe	Temporal lobe	Infratentorial	Basal ganglia
Grade 1	29.6	25.1	32.3	13.5	16.1
Grade 2	30.0	33.1	11.0	8.5	37.2
Grade 3	33.6	30.5	7.6	0.4	10.8

While the frontal lobe revealed the highest percentage of grade 3, diffuse lesions, grade 2 lesions were most frequently observed in the basal ganglia and grade 1 focal lesions within the temporal lobe, together indicating the different vulnerability of the cerebral vasculature of the respective region.

**Figure 2:**
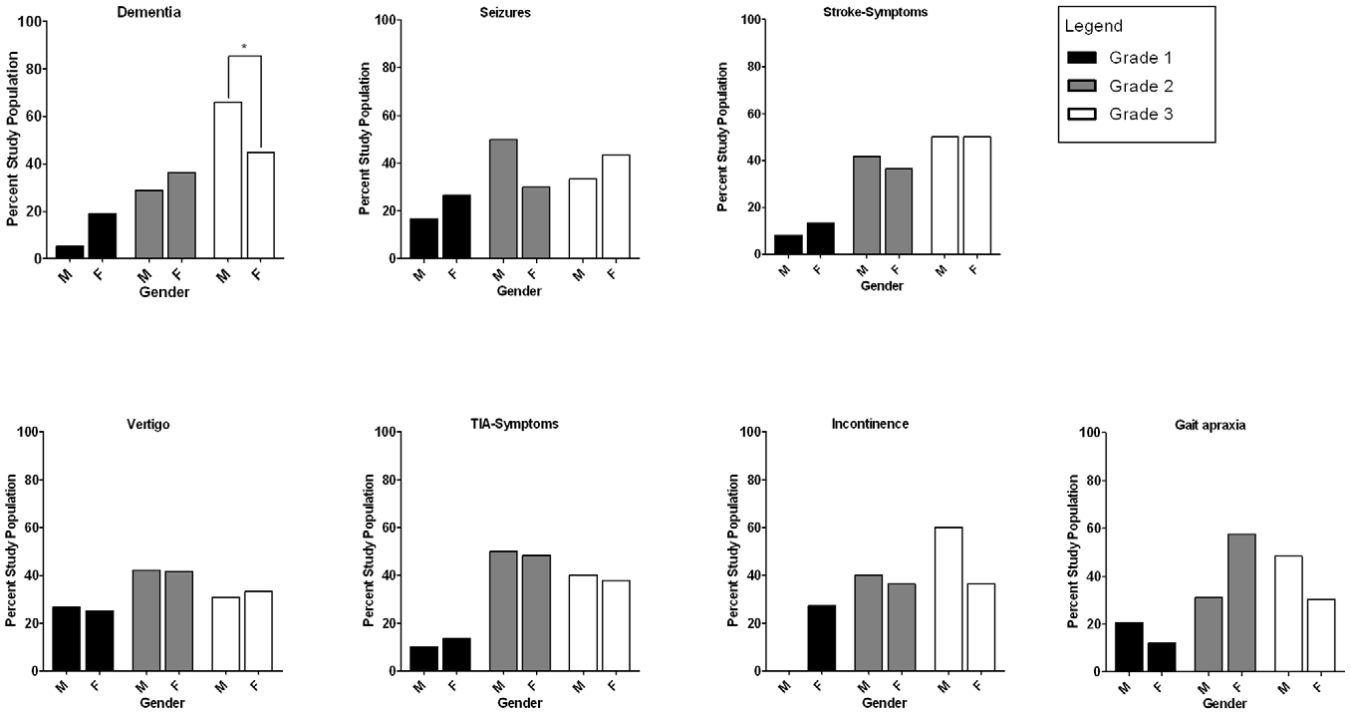
Gender and severity-dependent occurrence of neurological symptoms at admittance. Data from 223 patients, 98 males (*M*) and 125 females (*F*) were graded for lesion severity based on CT or MRI scans using the system described by Wahlund, et al., 2001 (Grade 1 =  focal lesions, 2 =  confluent lesions, 3 =  diffuse lesions). With the exception of progressive cognitive decline that occurred more frequently in male patients suffering from Grade 3 WML/lacunar infarcts, symptoms were about equally distributed across severity grades and gender (*p = n.s.,* Pearson's chi square).

The distribution of the lesion grades was analyzed according to gender and across clinical symptoms (Fig. 2). An almost equal lesion grade and gender distribution for most symptoms was found (Pearson's chi square = ns). Nevertheless, there were significantly more male patients that presented with grade 3 WMLs/lacunar infarct load and who had dementia compared to females with a similar WML/lacunar infarct lesion load.

### Risk Factors

**Figure 3:**
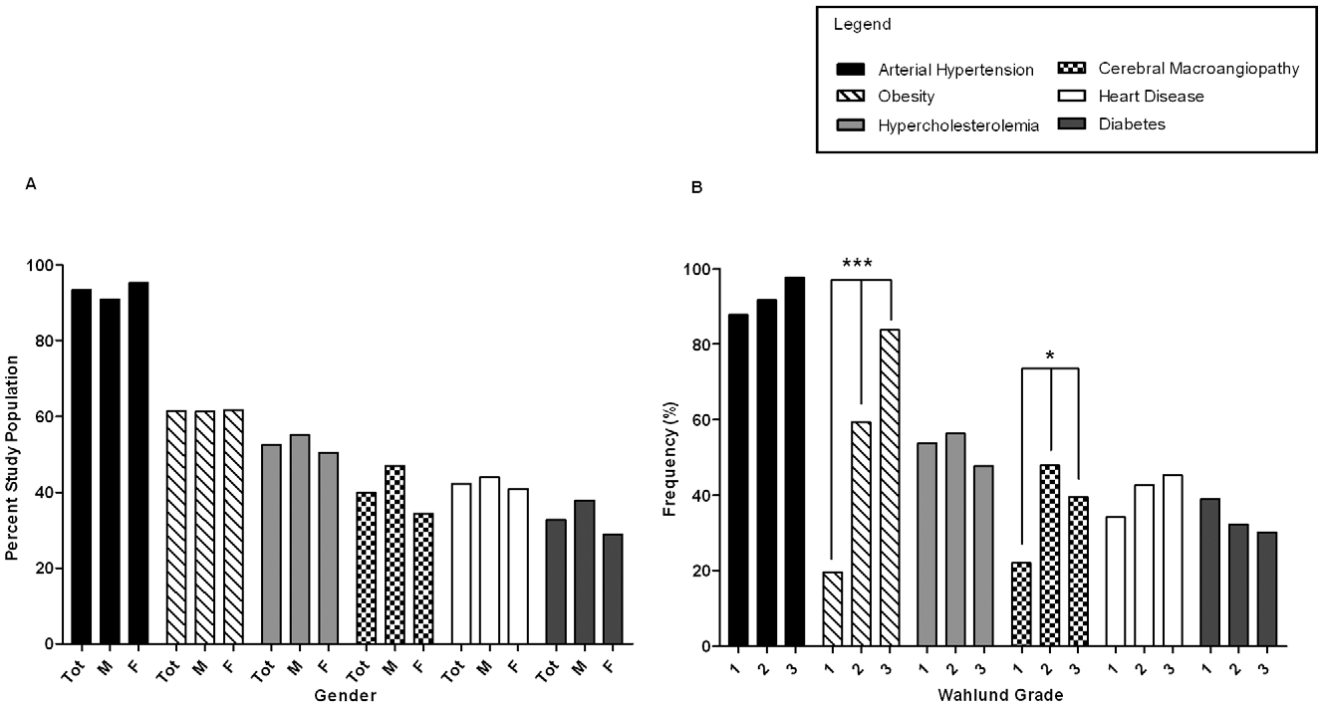
Frequency of vascular risk factors found in patients with cerebral microangiopathy. (**A**) The frequencies of vascular risk factors including arterial hypertension, obesity, hypercholesteremia, cerebral macroangiopathy, heart disease and diabetes are given here as percentages of total study population and broken down by gender. (**B**) The frequency of vascular risk factors in across different Wahlund grades (Wahlund, et al., 2001; Grade 1 =  focal lesions, Grade 2 =  confluent lesions, Grade 3 =  diffuse lesions). Occurrence of obesity (***p<.000, Pearson's chi square test) and cerebral macroangiopathy were unevenly distributed among the severity grades of WML and lacunar infarcts (*p = *.018, Pearson's chi square test).

Figure 3A shows the contribution of individual vascular risk factors to the development of WMLs/lacunar infarcts. Almost all patients suffered from arterial hypertension, followed by obesity, hypercholesterolemia and cerebral macroangiopathy. Heart disease and diabetes appeared in about 30–40% of patients. After correcting for the higher number of female patients, risk factors were equally distributed between genders. While hypercholesterolemia, heart disease and diabetes did not show a relation with WML/lacunar infarcts, the prevalence of vascular risk factors showed a stepwise and significant increase for obesity and cerebral macroangiopathy (Pearson`s chi square, Fig. 3B).

Combinations of risk factors arterial hypertension and obesity together, and the co-occurrence of arterial hypertension and hypercholesterolemia led to higher risk of WML/lacunar infarct development (57.8% and 50.7% respectively). There was a combined effect of cluster arterial hypertension and obesity, and this was related to severity grading (*p*<.000, Pearson's chi square).

### Clinical measurements and blood test results

White matter lesion grade and MAP, systolic and diastolic blood pressure showed strong associations (*p<*.000, One-way ANOVA). The mean BMI was elevated (*BMI_mean_  = *26.2) and strongly correlated with severity grading of white matter lesions (Table 3). In contrast, several other factors were unrelated to lesion grading. Thus, mean serum levels of glucose, and HbA1c had no effect on WML/lacunar infarct grades. Mean serum levels of cholesterol were slightly elevated. Triglycerides and LDL decreased, while HDL increased across the groups according to WML/lacunar infarct severity, however, there was no significant effect on WML/lacunar infarct grading. Similarly, mean serum levels of CRP, homocysteine and d-dimer-FSP were elevated, but showed no effect on WML/lacunar infarct grading (Table 3).

**Table 3:** Cardiovascular risk factors in relation to different WML/lacunar infarct grades.

		Wahlund Grade												
		Total			Grade 1			Grade 2			Grade 3			
	Norm	*n*	*M*	*SE*	*n*	*M*	*SE*	*n*	*M*	*SE*	*n*	*M*	*SE*	*p*
MAP (mmHG)^a^	<105	223	109.2	15.7	41	99.6	10.9	96	105.8	12.3	86	117.5	17.2	**<.000**
BP systolic (mmHg)^a^	<140	223	156.0	24.9	41	140.1	20.4	96	151.7	20.8	86	168.4	25.5	**<.000**
BP diastolic (mmHg)^a^	<90	223	85.8	13.6	41	79.6	9.0	96	82.9	11.3	86	92.0	15.3	**<.000**
BMI^a^	<25	223	26.2	3.2	41	23.9	2.1	96	25.8	3.2	86	27.7	2.9	**<.000**
Glucose (mg/dl)^a^	<120	218	118.3	39.4	39	125.4	44.3	95	113.5	30.6	84	120.4	45.3	.233
HbA1c (%)^a^	<6.5	115	6.0	1.0	23	6.0	1.1	48	6.0	0.7	115	6.0	1.0	.844
Cholesterol (mg/dl)^b^	<200	122	201.2	50.7	23	213.5	56.5	57	200.8	43.0	42	195.0	56.3	.989
Triglycerides (mg/dl)^b^	<150	114	126.5	66.4	19	125.0	64.0	55	133.4	77.0	40	117.6	50.5	.665
LDL (mg/dl)^b^	<150	122	124.8	42.8	22	131.3	41.4	58	124.7	35.2	42	121.6	52.6	.373
HDL (mg/dl)^b^	>40	121	54.5	17.0	22	58.0	19.9	58	53.9	15.9	41	53.6	17.1	.563
CRP (mg/l)^b^	<3	214	11.1	30.5	40	8.6	27.0	89	10.6	39.0	85	12.9	20.5	.742
Homocysteine (μmol/l)^b^	<10.7	14	73.0	58.0	2	49.7	55.6	8	66.2	57.0	4	98.1	67.1	.590
D-Dimers-FSP (mg/l)^b^	<0.5	49	1.4	1.2	7	0.94	0.8	24	1.6	1.5	18	1.2	0.7	.329

Cardiovascular risk factors have been calculated for a total mean of all patients and for each Wahlund grade (G1 =  Grade 1, G2 =  Grade 2, G3 =  Grade 3). Cardiovascular risk factor assessment included mean arterial blood pressure (MAP), systolic and diastolic blood pressure (BP), body mass index (BMI), as well as the serum determination of glucose, glycohemoglobin (HbA1c), cholesterol, triglycerides, high and low-density lipoproteins (HDL, LDL), C-reactive protein (CRP), homocysteine and D-dimers. Group differences were found for MAP, systolic and diastolic BP and BMI (all ***p<.000, univariate ANOVA). ^a^Norm values according to WHO. [5] ^b^Norm values equal to those of the Institute of Clinical Chemistry and Pharmacology at the University of Bonn [5].

## Discussion

We found that the most prevalent symptoms of CM were progressive cognitive decline, seizures and gait apraxia, followed by stroke or TIA related symptoms and vertigo. This confirms previous studies which relate silent stroke, [26] TIA, [27] gait disturbance, [5] incontinence [6] and cognitive decline [8] to WMLs and lacunar infarcts. Seizures and vertigo may simply have not been examined in depth in the context of CM in previous studies [28], [29]. It remains unclear how CM increases the risk of developing seizures in these patients, though it could be speculated that near-cortex WMLs/lacunar infarcts may also affect U-fibers, leading to a higher propensity for seizures as described for multiple sclerosis patients [30].

We did not find any gender effects for single clinical symptoms, nor for WMLs/lacunar infarct severity, or for the distribution of vascular risk factors.

The importance of the topography of WMLs/lacunar infarcts was strengthened by our results. Frontal lobe WMLs/lacunar infarcts led to more frequent presentation of progressive cognitive decline, seizures, gait apraxia, stroke-related symptoms, TIA, vertigo and incontinence. Parietooccipital WMLs/lacunar infarcts led to higher frequencies of TIA, seizures and incontinence. Basal ganglia WMLs/lacunar infarcts related to more complaints of gait apraxia, vertigo and incontinence. In the case of vertigo [31] and cognitive impairment, [32] the relations of symptoms to the topography are in line with previous reports.

As previously shown, we confirmed the effect of age on lesion severity. Patients in the two youngest age cohorts, 45–55 and 56–65 years, showed predominantly grade 1 lesions, while grade 3 lesions were almost absent. In strong contrast, this pattern reversed in older age cohorts (76–85 years): in addition to an overall increase in number of cases more severe grades (2 and 3) dominated. This older cohort also represented nearly half of the entire study population. This finding points to age as one major risk factor, as may be expected. It also indicates a window of opportunity up to the age of 65 in which progression from low to severe grade WMLs or lacunar infarcts may be positively influenced by risk factor treatment.

A new finding was obesity as an independent risk factor for development of CM. Age and raised systolic and diastolic blood pressure have previously been identified as potential risks, and were also found here [9], [12]. Several other potential CM risk-factors could not be confirmed in this study. Thus, diabetes was not identified as a risk factor for CM, even though the mean levels of HbA1c and serum glucose were increased in those patients [11], [33].

Furthermore, no relation of CM with serum levels of homocysteine, CRP and D-dimers were detectable. For homocysteine and d-dimers this could be due to the small number of cases having these laboratory parameters evaluated. Mean levels of all of these parameters were, however, elevated in CM patients. Lipids decreased across grades 1 to 3, although this finding was not statistically significant. The trend however speaks to a previous study suggesting a protective role of lipids in the progress of CM [34].

A limitation of this study may be the specific cohort (hospital based, only patients from the Department of Neurology), which may not be representative for all patients suffering from CM. Furthermore an overlap with other causes of small vessel disease (amyloid angiopathy, inherited, inflammatory, mixed dementia due to vascular degeneration and Alzheimer`s disease) cannot be ruled out completely, despite proper medical history taking, clinical and biochemical investigations. Further, there is likely an inherent bias in the sampling method due to the fact that medical investigations may have varied from patient to patient. In addition, not all patients underwent a CT and MRI-scan. We used a grading scale that is applicable for CT and MRIs, but both modalities do not detect equally well. While MRT is more sensitive for smaller lesions, CT is superior in detecting larger lesions which correlate better with symptoms [24].

## Conclusions

In conclusion, this study underlines the association of CM with progressive cognitive decline, stroke-related symptoms, gait apraxia and other neurological symptoms upon admission in a topographical manner, although the generalizability of this retrospective analysis may be limited. Importantly we found that, in addition to known risk factors such as aging and arterial hypertension, obesity increases the WML/lacunar infarct occurrence and severity. The higher incidence of seizures in our population deserves further investigation, as the mechanism underpinning this effect is unresolved. Future studies should focus on the importance of prevention of vascular risk factors on the pathogenesis of WML and lacunar infarcts.
